# Macular ischemia after intravitreal amikacin on patient with intraocular foreign body

**DOI:** 10.3205/oc000061

**Published:** 2017-03-24

**Authors:** Arief Kartasasmita, Susi Mona, Erwin Iskandar, Iwan Sovani, Djonggi Panggabean

**Affiliations:** 1 Faculty of Medicine, Universitas Padjadjaran/Cicendo National Eye Hospital, Bandung, Indonesia

## Abstract

**Background:** Although still used in third world countries, amikacin has a harmful effect to be used intravitreally.

**Purpose:** To report macular ischemia after an intravitreal injection of amikacin

**Methods:** A case report regarding a traumatized eye of a 26-year-old man that was injected intravitreally with amikacin due to intraocular foreign body endophthalmitis

**Results:** Angiography and OCT show macular ischemia due to amikacin toxicity.

**Conclusion:** The case reported here is to alert about the potential harmful effect of intravitreally injected amikacin despite its role as an accepted regimen for endohthalmitis cases.

## Introduction

Ocular trauma is the leading cause of monocular visual loss and a major cause of ocular morbidity. It is estimated that 500,000 blinding ocular injuries occur globally each year. The intraocular foreign body (IOFB) represents one form of open globe injury commonly described, especially in developing countries and rural areas. A variety of reports note that retained foreign bodies occur in 5–40% of all penetrating eye injuries [1], [2]. This is cause for concern as retained foreign bodies are associated with increased risk of endophthalmitis [3], [4]. The incidence of infectious endophthalmitis after penetrating injury with IOFB ranges from 0% up to 16.5% [5].

Broad-spectrum antibiotic intravitreal injection is the mainstay therapy following IOFB injury. To provide adequate Gram-negative and -positive bacterial cover in endophthalmitis cases, a currently accepted therapeutic regimen includes aminoglycosides [6]. In developing countries, aminoglycosides have become a favorable drug of choice due to their ease of access and reduced cost. However, these treatments are not without side effects: retinal vascular infarction is commonly described after gentamicin administration, and has been noted after use of amikacin [7]. While previously noted as a rare complication, the reported frequency of macular ischemia following amikacin intravitreal injection for the treatment of endophthalmitis has increased recently [6], [8]. This report describes a case of macular ischemia following intravitreal amikacin injection. 

## Case description

A 26-year-old man came to the emergency room in our tertiary eye hospital 1 day after his right eye was hit by a nail while hammering. The patient was referred from a district hospital with an IOFB in the right eye. He received an injection of anti-tetanus serum at initial admission. 

**Figure 1:**
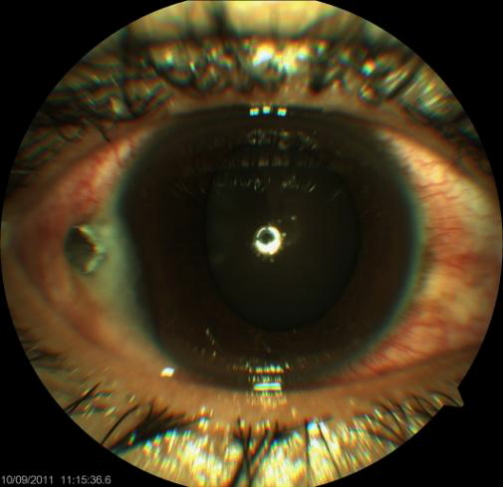
A nail has lodged in the right eye

The general examination was within normal limits. An ophthalmological examination was conducted; best corrected visual acuity (VA) was 6/5 in each eye, ocular motility was full in all directions, and digital intraocular pressures were normal for both eyes. The anterior segment of the right eye showed a hyperemic conjunctiva with a nail lodged at the temporal limbus (Figure 1 (Fig. 1)). Corneal edema was present, but other anterior segment findings were normal (round pupil, no synechiae or lens opacities, and anterior chamber formed with no flare or cells). The anterior segment of the left eye was within normal limits. Fundoscopic examination of the right eye revealed clear media with a visible foreign body (nail) in the vitreous, a round and sharp border of the optic disc, a flat retina, and good foveal reflex. The posterior segment of the left eye was within normal limits. 

The patient was diagnosed with an IOFB in the right eye. His treatment plan included intravenous cefotaxime injection (1 g) twice daily and hourly ofloxacin eye drop for the right eye in addition to being scheduled for IOFB extraction and intravitreal antibiotic (IVAB) injection. 

**Figure 2:**
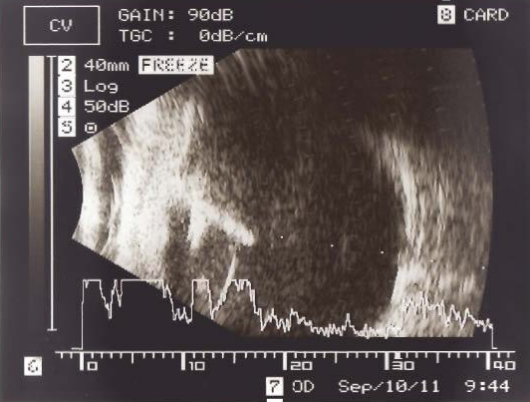
Ultrasonography showed an IOFB in the right eye

Ultrasonography examination showed an IOFB located in the limbus/pars plana that had penetrated into the vitreous cavity and posterior segment inflammation (Figure 2 (Fig. 2)).

The surgery was performed 6 hours after administered. The nail was lodged at the limbus (9 o’clock) and had penetrated into the vitreous cavity, contacting the edge of the lens. It was removed with forceps, and measured 9 mm in length. Anterior vitrectomy and vitreous tap were then performed around the wound and the vitreous contents were subjected to Gram staining and culture. The wound was sutured, and vancomycin (1 mg) and amikacin (0.2 mg) were injected intravitreally. The patient was also given oral paracetamol (500 mg) three times daily and prednisolone acetate eye drops six times daily for the right eye. 

**Figure 3:**
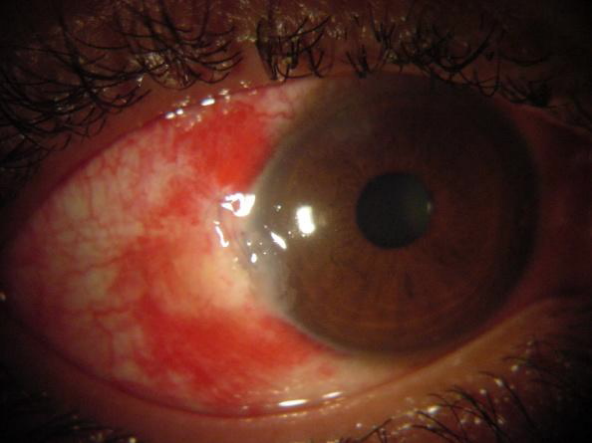
Anterior segment of the right eye after IOFB removal

On the first postoperative day, the uncorrected VA for the right eye was count fingers. Anterior segment examination showed a closed wound and two in-place sutures at the temporal limbus (Figure 3 (Fig. 3)). Corneal edema, a hyperemic conjunctiva, subconjunctival bleeding, and a moderate depth anterior chamber with inflammation were noted. As before, the pupil was round and no synechiae or lens opacities were observed. The posterior segment examination revealed clear media, a normal optic disc, a flat retina, and macular edema with decreased foveal reflex. Gram stain of the vitreous tap revealed the presence of Gram-positive cocci, while the KOH stain was negative. The previous therapies were continued with the addition of 48 mg of methyl prednisolone orally once a day. 

**Figure 4:**
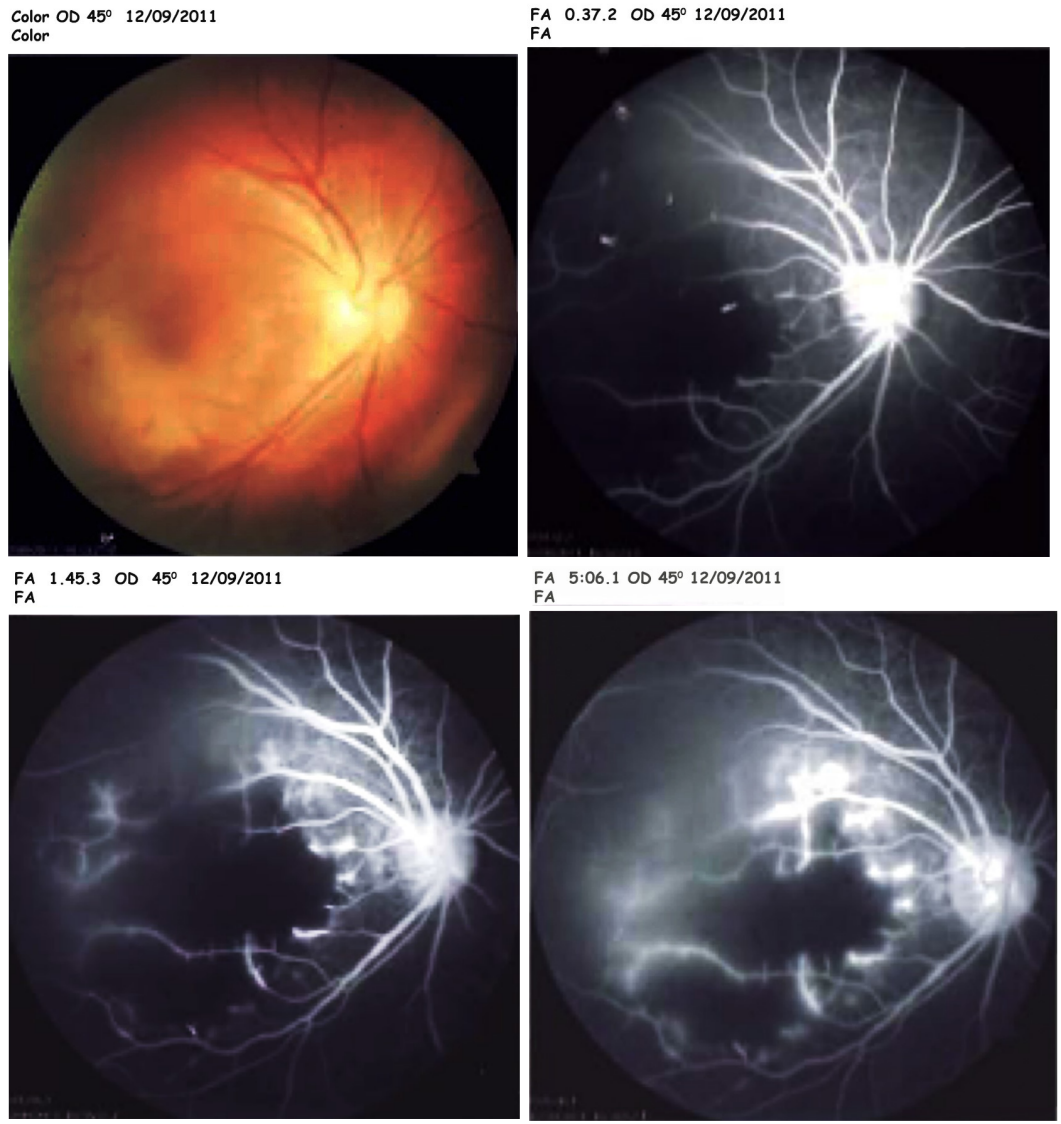
Angiography of the right eye showed macular ischemia

Angiographic examination of the right eye revealed hypofluorescence in the arteriovenous phase due to a vascular filling defect at the macula. Angiographic examination of the left eye was within normal limits. Based on the angiogram results, we diagnosed macular ischemia of the right eye (Figure 4 (Fig. 4)).

**Figure 5:**
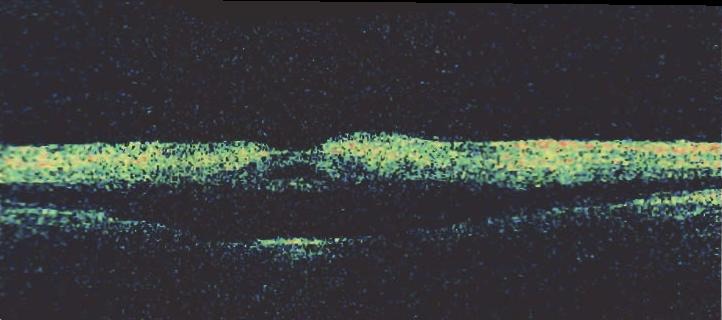
OCT of the right eye

Optical coherence tomography (OCT) examination of the right eye revealed increased retinal thickness with reduced intraretinal reflectivity, although the signal strength was ambiguous. OCT examination of the left eye was within normal limits (Figure 5 (Fig. 5)).

Three days after surgery, the uncorrected VA of the right eye was still count fingers, although there was a decrease in subconjunctival bleeding, corneal edema, and anterior chamber inflammation. The posterior segment examination continued to show macular edema. Previous therapies were continued with the addition of oral ciprofloxacin (750 mg) twice daily. The patient was discharged from the hospital with a review scheduled 1 week later.

## Discussion

Endophthalmitis prevention using prophylactic intraocular antibiotic injection in patients with penetrating injuries remains controversial. However, in cases where there is a high suspicion of injury from a contaminated foreign body or indeed early signs of endophthalmitis are present, prophylactic treatment with IVABs should be considered. Intraocular antibiotics should target a broad range of Gram-positive and -negative organisms. Vancomycin remains the drug of choice for treatment of Gram-positive infections. The aminoglycoside class of antibiotics is selected particularly for their Gram-negative coverage. However, caution should be exercised in their use because of reports of retinal vascular infarction following intravitreal injection [8]. IVABs were administrated in this patient because of concern that the IOFB may be contaminated, given the patient’s history of employment as a furniture maker. 

Cases of macular infarction secondary to administration of intraocular aminoglycosides have been observed after excessive intraocular doses while others have occurred after normal use [9]. There are several potentially toxic antibiotics in the aminoglycoside family including tobramycin and amikacin, with gentamicin having the greatest toxicity profile [10]. Following concerns about the risks of retinal toxicity caused by intravitreal gentamicin, many ophthalmologists now prefer intravitreal amikacin even though its use has also been associated with retinal toxicity [11]. Massive doses may result in early superficial and intraretinal hemorrhages, retinal edema, cotton wool patches, arterial narrowing, and venous beading [9]. In our patient, on the first day following IOFB extraction and IVAB injection we noted sudden decreased VA with the posterior segment examination revealing macular ischemia. This was subsequently confirmed by OCT and FFA examinations.

In an animal study, aminoglycoside toxicity occurred because this antibiotic affects primarily neurons and glia of the inner retina. This suggests that because this area has a high number of paramacular density cells, it is more susceptible to toxicity impairment. Other important factors include the gravitational forces of the antibiotic solution, which has a higher gravity than the vitreous or vitreous replacement compound. The latter was observed in studies using rabbits in which positioning of the eye on gentamicin injection caused toxic effects predominantly in the corresponding part of the retina [12].

Potential toxicity reactions of the retina are also subject to individual variation. Studies show that the risk of retinal toxicity is greater if drugs are injected towards the posterior pole with the needle pointed directly to the macula [8]. Moreover, higher injection frequencies may also increase the possibility of retinal toxicity [13]. Furthermore, intravitreal injections of aminoglycosides intended for the subconjunctival space that inadvertently miss may direct a toxic dose into the retina [12].

In our case, another etiological possibility for the macular edema observed is vancomycin toxicity from the initial combined amikacin-vancomycin injection. Studies on rabbits demonstrated that intravitreal vancomycin injection results in retinal toxicity and ERG abnormalities. However, this effect only occurred with large doses (5 and 10 mg) of vancomycin [12]. The doses of vancomycin used in this case (1 mg) are considered safe, given that no retinal toxicity has been observed in experimental rabbit eyes and no cases of human macular toxicity attributed to vancomycin at these doses have been reported. Based on previous reports, the intravitreal amikacin injection is strongly suspected as the main cause of ischemia in our patient. 

The overall prognosis for this patient is good because there is no systemic disorder caused by the trauma. The visual prognosis is poor, because the macular ischemia is an irreversible condition. 

Although still commonly used, especially in third world countries because of its availability and efficacy, we caution the use of amikacin as an intravitreal therapeutic agent given its potential for toxicity to the retinal tissues. Alternative and effective antibiotic agents are available with safer toxicity profiles.

## Notes

### Competing interests

The authors declare that they have no competing interests.
